# Whether innate immune together with genetic factor are involved in leukemic arthritis? A case report and review of the literature

**DOI:** 10.1097/MD.0000000000009919

**Published:** 2018-02-16

**Authors:** Danyi Xu, Guanhua Xu, Jin Lin

**Affiliations:** The First Affiliated Hospital, College of Medicine, Zhejiang University, Hangzhou, China.

**Keywords:** HLA-B27, innate immune, leukemic arthritis, monocytic leukemia

## Abstract

We report a case of leukemic arthritis (LA) of monocytic differentiation, which presented with spondyloarthritis-like symptoms and a positive human leukocyte antigen-B27, and discuss its potential mechanisms.

The patient was admitted because of pain in her right knee and lower back for 18 months. Magnetic resonance imaging showed diffuse hyperintense signal in the bilateral liac bones and bone marrow edema and synovitis in the right knee.

The diagnosis of acute monocytic leukemia and LA were concluded by bone marrow aspiration and flow cytometry of the synovial fluid.

The patient had poor response to nonsteroidal anti-inflammatory drugs. One week after she received chemotherapy, the symptoms were dramatically relieved.

For 5-year follow-up, she got clinical remission without suffering pain of the right knee and the lower back.

Leukemic arthritis is a rare manifestation of leukemia with unknown mechanism and may be the initial presentation of leukemia. The problem whether abnormal immune response of the neoplasitc monocytes together with hereditary factors contribute to the pathogenesis of LA in adult is raised from this case, which worth further research.

## 1 Introduction

Leukemic arthritis (LA) is a rare manifestation of leukemia in adult. Most of the reported cases of adult LA indicated that LA occurs in strong association with leukemias of monocyte differentiation. Abnormal innate immune responses take predominant role in inflammatory arthritis.

Altered innate immune system by the neoplastic monocytes may take part in the pathogenesis of LA. However, the pathogenesis of LA is not clear, whether other factors like genetic factors take part in the process of LA were not mentioned in literature. We herein report the findings of another case of LA associated with monocytic leukemias, which presented with spondyloarthritis (SpA)-like symptoms and a positive human leukocyte antigen (HLA)-B27.

## 2 Case report

A 66-year-old woman presented to our rheumatology clinic in November, 2012, complaining of pain in her right knee and lower back without any other discomfort for 18 months. She was diagnosed of osteoarthritis (OA) in her right knee for the recent half year. The symptoms of pain and swelling in her right knee were aggravated over the preceding month accompanying with a lower back pain. On physical examination, the right knee was swollen and tender to palpation with decreased motion range; the sacroiliac joints were bilaterally tender on palpation. Her son had a history of ankylosing spondylitis for decades with a positive HLA-B27.

**Figure 1:**
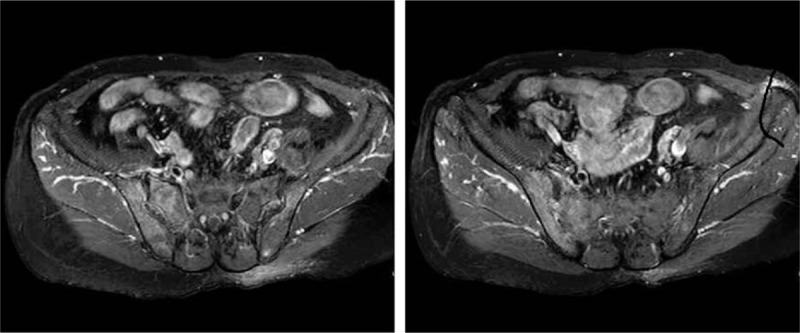
Magnetic resonance imaging (MRI) of the sacroiliac joints showed diffuse hyperintense signal on gadolinium-enhanced, T1-weighted, fat-saturated images in the bilateral liac bones which indicate abnormal bone marrow proliferation.

**Figure 2:**
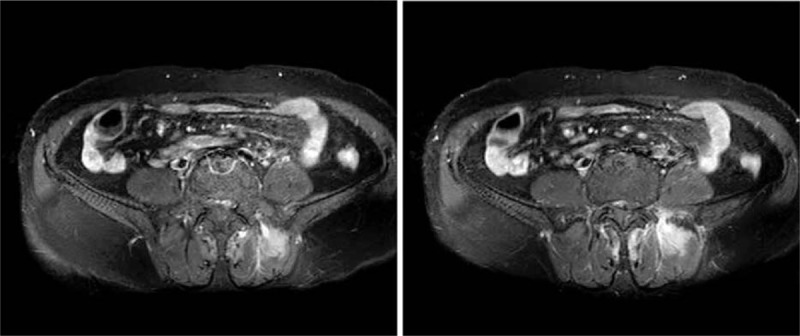
Magnetic resonance imaging (MRI) of the sacroiliac joints showed diffuse hyperintense signal on gadolinium-enhanced, T1-weighted, fat-saturated images in the left erector spinae muscles which indicate reactive myofascitis.

**Figure 3:**
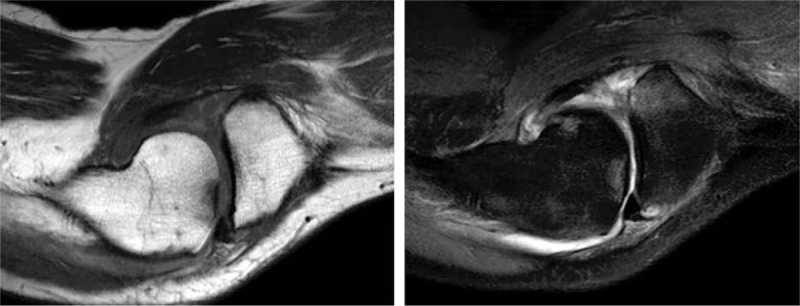
Magnetic resonance imaging (MRI) of the right knee showed bone marrow edema and synovitis.

**Figure 4:**
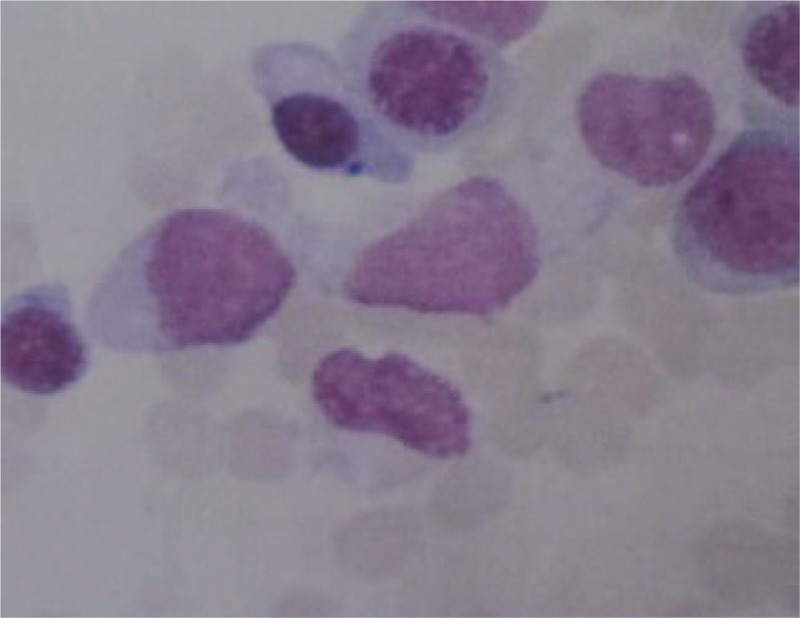
A bone marrow aspiration and biopsy sample showed marked monocytosis including many monoblasts and promonocytes (66%). Wright-Giemsa staining; magnification, ×1000.

The level of serum high sensitive C-reactive protein (CRP) was 89.2 mg/L and the erythrocyte sedimentation rate was 114 mm/h. The level of immunoglobulin G was 1760.0 mg/dL, complement C3 was 168.0 mg/dL, and complement C4 was 45.6 mg/dL. HLA-B27 was positive, whereas the rheumatoid factor, anticyclic citrullinated peptide antibodies, and antinuclear antibody were all negative. A computed tomography scan of the sacroiliac joints was performed without any manifestation of sacroiliitis. Magnetic resonance imaging (MRI) of the sacroiliac joints showed diffuse hyperintense signal on gadolinium-enhanced, T1-weighted, fat-saturated images in the bilateral liac bones and the left erector spinae muscles which indicate abnormal bone marrow proliferation and reactive myofascitis (Figs. 1 and 2). MRI of the right knee showed bone marrow edema and synovitis (Fig. 3). The white blood cell count was 7.5 × 10^9^/L, with a differential count of neutrophils (46.7%), lymphocytes (20.1%), and monocytes (22.6%). The hemoglobin was 97 g/L, and the platelet count was148 × 10^9^/L. Blast cells (4%) were detected in peripheral blood smears. A bone marrow aspiration and biopsy sample showed marked monocytosis including many monoblasts and promonocytes (66%) (Fig. 4). Flow cytometry of the bone marrow aspiration sample revealed a group of primitive myeloid cells (10.9%) which were positive for CD13, CD17, CD33, and HLA-DR; a large population of promonocytes (56.85%) was also identified which expressed CD11b, CD13, CD14 (partial), CD15, CD33, CD35, CD56 (partial),CD64, CD65s, and HLA-DR. A right knee aspiration yielded a yellow, mild cloudy synovial fluid. Synovial fluid cultures were negative. Flow cytometry of the synovial fluid sample revealed a group of monoblasts and promonocytes which were positive for CD2, CD11b, CD13, CD14, CD15, CD33, CD35, CD64, CD65s, and HLA-DR, which was similar to the findings of the bone marrow sample. The diagnosis of acute monocytic leukemia (AMoL) and LA was made definitely.

Diclofenac sodium 75 mg qd was given for 2 weeks, but she had poor response to it. One week after she received chemotherapy (Idarubicin 20 mg d1 10 mg d2-3; Cytarabine 75 mg q12 h d1-7), her joint pain was dramatically relieved, and inflammatory biomarkers were reduced to normal range (CRP was 5 mg/L, ESR was 12 mm/h). For 5 years follow-up, she got clinical remission without suffering pain of the right knee and the lower back.

## 3 Discussion

Leukemic arthritis, a rare manifestation of leukemia, may be the initial presentation of leukemia. LA is defined as joint pain and swollen in association with peripheral blood or bone marrow leukemia after other causes of arthritis have been excluded.^[1]^ Large joints are most commonly involved, and the joint pain is often severe with poor response to nonsteroidal anti-inflammatory drugs. Synovial fluid examination is critical for diagnosis of LA.

Synovial infiltration of the leukemic cells may be the predominant mechanism of LA. Acree et al^[1]^ firstly reported the association of adult LA and leukemias of monocyte differentiation. They found that their 4 cases and most of the prior reported cases of adult LA were all monocytic leukemia, indicating that adult LA occurs in strong association with leukemias of monocyte differentiation. An association of LA and pre-existing arthritis of OA or rheumatoid arthritis (RA) was also found in their reported cases. Monocytes-attracting cytokines like tumor necrosis factor α (TNF-α) play a significant role in both OA and RA, which mediate and increase monocytes migration into the inflamed joint space. They hypothesized that^[1]^ leukemic monocytes, like their benign counterparts, may migrate to sites of inflamed joints in response to monocytes-attracting cytokines, which may be critical in the mechanism of LA. However, question which remained unanswered is why do not all monocytic leukemias migrate to arthritic joints? Apart from previous history of arthritis and monocytes-attracting cytokines, there should be other associated factors. In our case, the old woman presented with new-onset SpA mimic symptoms with a positive HLA-B27, eventually diagnosed of LA. The question is whether there is an association among monocytic leukemias, HLA-B27, and LA, or just a coincidence.

Apondyloarthritis is a chronic inflammatory disease with a strong association of HLA-B27.^[2]^ HLA-B27 is involved in the occurrence of arthritis. The heavy chain of HLA-B27 molecules can be found on the cell surface and does not require the routine binding to the peptides of the β2 microglobulin or the classical HLA-B27 pathway. These free heavy chains, as stable dimers, can be combined with the locus-specific receptors of natural killer cells and T lymphocytes, which may then induce arthritis.^[3]^ On the contrary, HLA-B27 heavy chain, tend to be misfolded or unfolded inside the endoplasmic reticulum, combined with molecular chaperone, activates some of the process of the endoplasmic reticulum, known as the unfolded protein response (UPR). In SpA, UPR activates the pattern recognition receptors, which then produce a large number of proinflammatory factors, especially interleukin (IL)-23, by innate immune cells.^[4]^

In recent investigations, SpA was considered to be an autoinflammatory disease driven mainly by abnormal innate immune responses rather than by T-cell and/or B-cell autoreactivity.^[5]^ Tissue macrophages are the predominant innate immune cells, which are derived from monocytes in an inflammatory condition. In RA and SpA, when innate immune altered, macrophages heavily infiltrate the synovial tissues in inflammatory arthritis and also produce abundant key proinflammatory cytokines (TNF-α, IL-1, IL-23, IL-17) in the pathogenesis of RA and SpA.^[6,7]^ There may be certain relationship between the altered innate immune responses and the nonantigen-presenting functions of HLA-B27 including the induction of UPR. In the HLA-B27/Hub2m transgenic rats, ER stress as a result of HLA-B27 up-regulation increased the Toll-like receptor-induced IL-23 production by bone marrow-derived macrophages.^[8]^ The genetic background and altered innate immune system are considered to be the key factors in the pathogenesis of SpA.

The woman in our case was HLA-B27 positive, but she did not present symptoms of SpA until she suffered AMoL. We suppose her innate immune response was altered while the onset of AMoL by the neoplastic monocytes with abnormal quantitative and qualitative properties. We hypothesize that the altered innate immune response, together with the genetic factor HLA-B27, might play an important role in the pathogenesis of LA in our case.

## 4 Conclusions

In summary, LA is a rare manifestation of leukemia and may be the initial presentation of leukemia. LA is strongly associated with monocytic leukemias in adult, possibly owing to the important role of monocytes in the pathogenic mechanism of arthritis. The hypothesis that abnormal immune response of the neoplasitc monocytes, together with genetic factors, may contribute to the pathogenesis of LA in adults is worth further investigation.
